# Effects of *Allium fistulosum* L. (Green Onion) Root and *Avena sativa* L. (Oat) Mixtures (WCO31) on the Height of Children: A Multi-Center, Randomized, Double-Blind, Placebo-Controlled Clinical Trial

**DOI:** 10.3390/nu18091326

**Published:** 2026-04-22

**Authors:** You-Jin Kim, Do-Yeon Kim, Seong-In Cheong, Hye Jeong Yang, Min Jung Kim, Hyun-Jun Jang, Myung-Sunny Kim, Dai Ja Jang, Nu-Ri Ha, Seul-Ki Kim, Min-Hwan Bae, Jong-Cheon Joo, Soo-Jung Park

**Affiliations:** 1 WELLREST Co., Ltd., 119 Je3sandan-ro, Jecheon 27115, Republic of Korea; gagahoho@wellrest.co.kr (D.-Y.K.); seongin@wellrest.co.kr (S.-I.C.); 2 Precision Nutrition Research Group, Korea Food Research Institute, 245 Nongsaengmyeong-ro, Iseo-myeon, Wanju-gun 55365, Jeollabuk-do, Republic of Korea; yhj@kfri.re.kr (H.J.Y.); kmj@kfri.re.kr (M.J.K.); hyunjun@kfri.re.kr (H.-J.J.); truka@kfri.re.kr (M.-S.K.); djjang@kfri.re.kr (D.J.J.); 3 Department of Food Biotechnology, University of Science & Technology, 217 Gajeong-ro, Yuseong-gu, Daejeon 34113, Republic of Korea; 4 Department of Systems Biology, College of Life Science, Yonsei University, 50 Yonsei-ro, Seodaemun-gu, Seoul 03722, Republic of Korea; omphalos0911@gmail.com; 5 ERCOHS Agricultural Corporation Co., Ltd., 329 Doandong-ro, Yuseong-gu, Daejeon 34195, Republic of Korea; ksk@ercohs.com (S.-K.K.); bmh@ercohs.com (M.-H.B.); 6 Department of Constitutional Medicine, Wonkwang University Jeonju Korean Medicine Hospital, 99 Garyeonsan-ro, Deokjin-gu, Jeonju 54887, Jeollabuk-do, Republic of Korea; 7 Department of Sasang Constitutional Medicine, College of Korean Medicine, Woosuk University, Jeonju 55338, Republic of Korea

**Keywords:** green onion, *Allium fistulosum* L., oat, *Avena sativa* L., height growth, clinical trial

## Abstract

**Background/Objectives**: Following prior in vitro and in vivo investigations on the bone health benefits of green onions and oats, we aimed to assess the effects of WCO31, *Allium fistulosum* L. (green onion) root and *Avena sativa* L. (oat) mixtures, on height growth and safety. **Methods:** This multi-center, randomized, double-blind, placebo-controlled study included 150 children aged 6–8 years (75 males and 75 females) who fell between the 3rd and 50th percentiles of the Korean National Growth Charts but had not yet developed secondary sexual characteristics. They were randomly assigned to receive daily oral administration of WCO31 (1.2 g/day) or a placebo for 24 weeks. For efficacy analysis, height, growth rate, growth rate standard deviation score (SDS), height SDS, and growth-related parameters were measured. To evaluate the safety of the intervention, several safety parameters (including the incidence of adverse events, laboratory tests, and vital signs) were monitored. **Results:** The WCO31 group demonstrated significantly superior outcomes, including height, growth rate, growth rate SDS, height SDS, and height-for-age Z-score, than the placebo group (all *p* < 0.001). Moreover, no safety-related concerns were identified. **Conclusions:** WCO31 positively influences height growth and demonstrates a favorable safety profile, with no observable adverse effects. This study provides the first clinical evidence supporting growth enhancement using natural extracts, suggesting that WCO31 could serve as a cost-effective, safe, and accessible complementary strategy for promoting child growth.

## 1. Introduction

Bone tissue formation and growth peak during childhood and adolescence [1,2]. Bone development, along with bone modeling and remodeling, is affected by growth hormone (GH), insulin-like growth factor-1 (IGF-1), and sex steroid hormones, such as estrogen and androgens [3]. Bone modeling and remodeling occur throughout life [4]; however, skeletal growth is usually almost completed during adolescence and determines the final height [5]. There is an increasing demand for GH prescriptions and related functional foods for children. The demand for HT042, which was approved by the Ministry of Food and Drug Safety in 2014 to support physical growth in young children, has steadily increased, reaching over 60 billion won in 2021 [6]. Additionally, statistics from the Health Insurance Review and Assessment Service indicate a consistent increase in the prescription of GH by medical institutions over the past 5 years (2018–2022) [7]. However, reports of adverse events (AEs) associated with GH injections have also increased, raising concerns regarding the abuse and safety of GH prescriptions [7]. Accordingly, the MFDS reported that GH injections are not intended to increase height; rather, they are indicated for children with GH secretion disorders, and caution is needed to prevent misuse and manage side effects [8].

Many studies have been examined various factors affecting bone health and skeletal growth, including heredity, nutrition, exercise, and environment [9,10,11,12,13,14]. Proia et al. [9] suggested that the optimal time for physical activity to affect skeletal growth depends on the stage of maturity in children and adolescents and sex. Additionally, consuming an appropriate balance of macronutrients (protein, fat, and carbohydrates) and micronutrients (vitamin D and phosphorus) is essential in conjunction with calcium intake. These nutrients collectively support calcium metabolism and absorption, thereby increasing bone mineral density (BMD) and promoting optimal skeletal development [10,11,12]. In addition to insufficient nutrient intake, unsanitary environments can cause stunted growth and cognitive dysfunction in children [13].

According to the Korean medical book *Donguibogam*, the white part of the green onion, including its root, called “chongbaek,” relieves cold symptoms and promotes blood circulation [14]. Modern studies have further established that green onion (*Allium fistulosum* L.) is rich in organosulfur compounds and flavonoids, which possess potent antioxidant and anti-inflammatory properties [15,16]. However, research on the efficacy of *A. fistulosum* L., compared with that on garlic and onion (which belong to the same *Allium* genus), remains relatively limited. In addition, oat (*Avena sativa* L.) is a widely consumed grain and is known for its diverse nutritional profile. In particular, *β*-glucan, which is abundant in oats, has been demonstrated to exert anti-obesity, anti-hypertensive, and anti-diabetic effects by lowering serum triglyceride and cholesterol levels [17,18,19].

Despite these broad physiological benefits, some in vitro and in vivo studies have specifically indicated that green onion roots contribute to longitudinal bone growth [14,20]. In vitro, green onion bulb extract has been shown to significantly increase alkaline phosphatase (ALP) activity and calcium concentration in osteoblast-like cell lines, such as MC3T3-E1 and MG-63, suggesting its role in promoting bone formation [14]. In vivo, C57BL/6 mice fed a diet supplemented with these extracts ad libitum showed improvements in BMD and bone mineral content (BMC) of the femur and tibia [14]. Furthermore, another study confirmed that weaning rats provided with oat and green onion root extracts exhibited increased serum GH concentrations and longitudinal growth of the femur and tibia [20]. In both animal models, these extracts demonstrated positive trends in bone development similar to those observed in the positive control groups administered GH, suggesting their potential role in supporting skeletal health. Similarly, recent phytochemical studies have elucidated the mechanisms by which oat-derived compounds influence bone metabolism. Treatment with novel secondary metabolites isolated from oat seedlings has been shown to stimulate osteoblast differentiation in MC3T3-E1 cells, as evidenced by increased ALP activity and mineralized nodule formation. These effects are mediated through the upregulation of key osteogenic markers, including Runx2, osteocalcin, and type I collagen [21]. Collectively, these findings suggest that the bioactive constituents of both green onion and oat may synergistically support skeletal health by enhancing osteoblast activity and bone mineralization. However, to the best of our knowledge, no clinical trials have yet investigated the effects of these materials on human bone growth.

Therefore, we aimed to examine the effects of *A. fistulosum* L. root and *A. sativa* L. mixtures (WCO31) on height growth in children between the 3rd and 50th percentiles of the Korean National Growth Charts who had not yet developed secondary sexual characteristics.

## 2. Materials and Methods

### 2.1. Study Design

This 24-week, multi-center, randomized, double-blind, placebo-controlled clinical trial was conducted at Wonkwang University Jeonju Korean Medicine Hospital, Jeonju, Republic of Korea, and Woosuk University Korean Medicine Hospital, Jeonju, Republic of Korea, in accordance with the principles of the Declaration of Helsinki (2013). The study protocol was approved by the Institutional Review Boards of Wonkwang University Jeonju Korean Medicine Hospital (IRB approval number: WUJKMH-IRB-2023-0013) and Woosuk University Korean Medicine Hospital (IRB approval number: WSOH IRB H2312-03). This study was registered with the Clinical Research Information Service (CRIS; http://cris.nih.go.kr) (Clinical Trial No.: KCT0009726, approval date: 21 August 2024).

### 2.2. Study Participants

Participants were recruited based on strict eligibility criteria to ensure a homogeneous study population. Briefly, healthy children aged 6–8 years at the point of initial screening with heights in the 3rd–50th percentile range and no secondary sexual characteristics were included. The comprehensive inclusion criteria and 16 specific exclusion criteria, including detailed medical history and laboratory thresholds, are summarized in Table 1. All procedures were conducted after obtaining voluntary written informed consent from both the participants and their legal guardians.

### 2.3. Sample Size Determination

To determine the required sample size, the expected mean increases in height were estimated at 3.78 cm for the intervention group and 2.91 cm for the placebo group. The standard for these height changes was assumed to be 1.61 cm (d = 0.87, σ = 1.61). The target sample size was set at 54 individuals per group, ensuring 80% power (β = 0.2, Z_β_ = 0.84) to detect a statistically significant difference at a 5% alpha level (α = 0.05). Assuming a balanced 1:1 randomization ratio (k = n_t_/n_c_ = 1), the calculation was performed using the following mathematical model:

$$n=\frac{\left(1+\frac{1}{k}\right)\sigma^{2}{\left(Z_{\frac{\alpha }{2}}{+Z}_{\beta }\right)}^{2}}{d^{2}}=54$$

To enhance statistical robustness and ensure adequate power, the target sample size was initially established at 56 participants per arm. The final sample size was increased to 80 participants per group to account for a potential attrition rate of 30%. Participants were randomly allocated in a 1:1 ratio. To minimize potential sex-related bias, stratified block randomization was performed by sex (male and female) using the SAS software (version 9.4; SAS Institute, Cary, NC, USA). This approach ensured that sex-based differences were adequately balanced across study groups. For allocation concealment, an independent statistician not involved in participant recruitment or assessment generated the randomization sequence. The randomization codes were maintained in sequentially numbered, opaque, sealed envelopes to ensure that the assignment remained unpredictable to the clinical investigators until the point of enrollment. Although the master randomization code was maintained by the study sponsor, individual participants were assigned codes strictly in the order of enrollment by independent clinical investigators at each site. All investigators and participants remained double-blinded throughout the study. Unblinding was permitted only in emergency situations where knowing the specific treatment was essential for participant safety.

### 2.4. Study Procedures

Study participants were selected based on a comprehensive screening assessment performed after written informed consent was obtained. Individuals who fulfilled all eligibility criteria were subsequently enrolled in a 24-week clinical intervention. Following the initial screening, eligibility was re-confirmed at the baseline visit (week 0) that occurred within 4 weeks of the screening assessment. Only those who consistently met all inclusion criteria were formally enrolled in the clinical trial. Subsequently, the participants were randomly allocated to either the intervention or placebo group, after which baseline evaluations were conducted. Throughout the 24-week intervention period, participants received either WCO31 (1.2 g/day) or placebo once daily. Follow-up visits, during which clinical efficacy, vital signs, concomitant medication use, changes in health status, and AEs were rigorously monitored, were scheduled at 12-week intervals. Additionally, AEs occurring during or after the intervention period were monitored and recorded regardless of their causality. If an AE was deemed related to the sample intake, the intervention was immediately discontinued. Following the final dose or early termination visit, the investigator performed supplementary follow-up observations as clinically indicated to ensure participant safety. Participant compliance was monitored by evaluating residual samples returned at every scheduled visit. Compliance was determined by calculating the percentage of doses administered relative to the required number of doses according to the study protocol.

### 2.5. Sample Preparation

*A. fistulosum* L. roots and *A. sativa* L. were extracted separately with hot water. Subsequently, the samples were freeze-dried and pulverized. The mixture (WCO31) was prepared by mixing the respective powders at a ratio of 1:10 (*A. fistulosum*: *A. sativa*).

The WCO31 sample was prepared by mixing 40% WCO31, 20% anhydrous crystalline glucose, 3% anhydrous citric acid, 4% berry-mixed flavor powder, 1% enzymatically processed stevia, 5% trehalose, 24% crystalline fructose, and 3% blueberry flavor powder. The placebo sample was prepared by mixing 24% maltodextrin instead of WCO31, 40% anhydrous crystalline glucose, 3% anhydrous citric acid, 4% berry-mixed flavor powder, 1% enzymatically processed stevia, 5% trehalose, 20% crystalline fructose, and 3% blueberry flavor powder. The two samples were indistinguishable in terms of color, smell, and shape.

### 2.6. Statistical Analysis

Statistical analyses were performed using SAS software (version 9.4; SAS Institute, Cary, NC, USA), with statistical significance defined as *p* < 0.05. The per-protocol (PP) set, consisting of participants who completed the study without major protocol deviations, was used for the primary efficacy analysis. Safety and intention-to-treat (ITT) analyses were performed on the Safety and ITT sets, respectively (in this study, both sets consisted of the same population). To address missing data within the ITT set, multiple imputation (MI) with 20 iterations was employed to provide more robust estimates than simple imputation techniques. Detailed results of this comprehensive ITT analysis are available in the Supplementary Materials.

#### 2.6.1. Efficacy Analysis

The primary efficacy outcome (height) and secondary efficacy parameters—including growth rate, growth rate SDS (standard deviation score), height SDS, growth-regulating factors (IGF-1, IGF-1/IGFBP-3 ratio, and GH), bone-related indicators (bone age and osteocalcin), and growth-related Z-scores (weight-for-height Z [WHZ]; height-for-age Z [HAZ])—were statistically compared between the WCO31 and placebo groups. Within-group variations between pre- and post-intervention periods were examined using paired *t*-tests or linear mixed models (LMMs), while between-group differences in 24-week changes from baseline were primarily analyzed using independent *t*-tests or analysis of covariance (ANCOVA).

To account for potential confounding factors, ANCOVA was primarily used for the PP analysis to compare changes from baseline between groups with adjustments for treatment compliance or baseline values as covariates. For the ITT analysis, LMMs were employed to incorporate MI results and to account for the longitudinal nature of the data (0, 12, and 24 weeks). Effect sizes and their 95% confidence intervals (CIs) were derived from the differences in Least Squares (LS) means in the adjusted models.

To ensure consistency and account for multiple comparisons in post hoc analyses, *p*-values and 95% CIs were adjusted using the Bonferroni method where appropriate. To further evaluate the robustness of the treatment effect and address potential baseline imbalances, a sensitivity analysis was performed using a median-split subgroup approach. Participants were stratified into lower and upper subgroups based on the median value of the imbalanced baseline variable and analyzed using the same LMM framework with adjustments for compliance and baseline values.

#### 2.6.2. Safety Analysis

We summarized AEs reported throughout the intervention period as frequencies and percentages for the total population and by the study group. Variations in occurrence patterns between the intervention and placebo groups were assessed using the chi-square test or Fisher’s exact test. Between- and within-group comparisons were conducted for laboratory tests (complete blood count, biochemistry tests, urinalysis, sex hormone tests) and vital signs (systolic blood pressure, diastolic blood pressure, and pulse). To handle missing data in these safety variables, 20 iterations of MI were performed using the fully conditional specification method. LMMs were then employed to compare within- and between-group mean changes and to evaluate the significance of between-group differences in changes by analyzing the group-by-visit interaction effect. All resulting estimates from the imputed datasets were pooled according to Rubin’s rules to derive the final *p*-values. In cases where the assumption of normality was not met, non-parametric tests—including the Wilcoxon signed-rank test for within-group comparisons and the Mann–Whitney U or Kruskal–Wallis tests for between-group comparisons—were employed.

## 3. Results

### 3.1. Study Participants

Overall, 162 volunteers (potential study participants) provided written informed consent. Of these, 160 individuals who satisfied all predefined inclusion and exclusion criteria were enrolled in the study. These participants (*n* = 80 per group) were assigned to receive either WCO31 or placebo. The treatment regimen consisted of a single daily dose over a 24-week intervention period.

**Figure 1:**
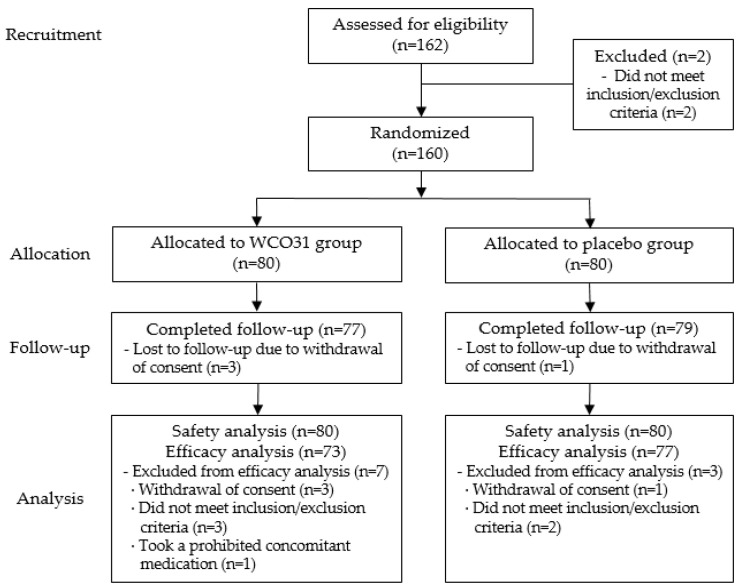
CONSORT flowchart illustrating the screening, randomization, intervention assignment, and final analysis of study participants.

Among the 160 randomized participants, 10 were excluded from the PP set for the following reasons: five individuals failed to satisfy the eligibility criteria (WCO31, *n* = 3; placebo, *n* = 2), four withdrew their consent (WCO31, *n* = 3; placebo, *n* = 1), and one used prohibited concomitant medication. Finally, 150 participants completed the study according to the protocol and were included in the analysis (Figure 1).

### 3.2. Characteristics of Study Participants

A total of 160 participants were equally allocated to either the WCO31 or placebo group (*n* = 80 per group), each comprising 40 males and 40 females. The mean age was 6.69 ± 0.76 years in the WCO31 group and 6.93 ± 0.79 years in the placebo group; no significant difference in age was observed between the two groups (*p* > 0.05).

At baseline, the mean height was 118.90 ± 5.26 cm and 120.29 ± 5.41 cm in the WCO31 and placebo cohorts, respectively. The mean weight was 22.20 ± 3.67 kg in the WCO31 group vs. 23.17 ± 4.62 kg in the placebo group. The mean body mass index was recorded as 15.64 ± 1.86 kg/m^2^ and 15.90 ± 2.15 kg/m^2^ in the WCO31 and placebo groups, respectively. The standard growth chart values were 28.99 ± 14.59 in the WCO31 group and 28.44 ± 14.90 in the placebo group. No significant differences were observed between the two groups (*p* > 0.05), indicating baseline homogeneity. Other variables, including blood pressure, pulse, birth length, birth weight, and outdoor activity time, also showed no significant differences between the groups (*p* > 0.05) (Table 2).

### 3.3. Compliance

Treatment compliance differed significantly between the two groups (94.97 ± 2.97% and 93.80 ± 3.71% in the WCO31 and placebo groups, respectively; *p* = 0.036). Therefore, the results adjusted for compliance are also presented in the efficacy and safety analyses.

### 3.4. Efficacy Analysis

A total of 150 participants were included in the PP set (73 and 77 in the WCO31 and placebo groups, respectively).

#### 3.4.1. Efficacy Analysis at Baseline

The growth rates of the WCO31 and placebo groups were significantly different (*p* < 0.001). Consequently, all subsequent efficacy outcomes are reported as adjusted values to account for this initial imbalance. No significant between-group differences in the other efficacy variables were observed at baseline (*p* > 0.05) (Table 3).

#### 3.4.2. Primary Efficacy Analysis

**Figure 2:**
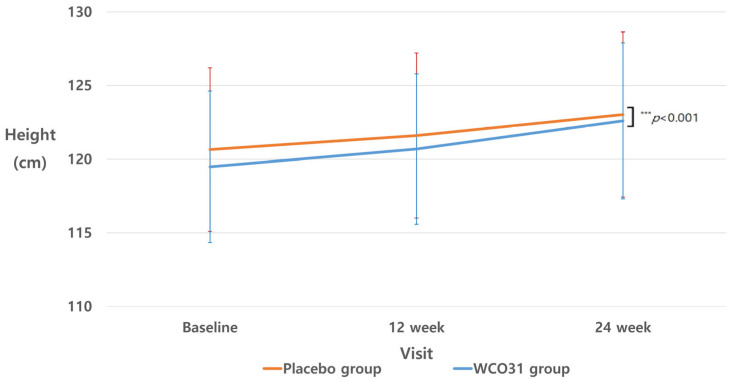
Height changes by visit over 24 weeks.

Primary efficacy evaluations of height were conducted at baseline and during follow-up visits at weeks 12 and 24. Table 4, Table 5 and Table 6 and Figure 2 summarize the findings of both the between-group and within-group statistical analyses across these intervals.

A significant difference was observed in height increments from baseline to week 12 between the two groups (*p* = 0.032). Specifically, the WCO31 group exhibited a greater increase of 1.20 ± 0.70 cm, whereas the placebo group showed a gain of 0.95 ± 0.69 cm. This difference remained significant even after adjusting for treatment compliance (*p* = 0.032). Additionally, regarding between-group changes, both groups exhibited significant increases in height from baseline to week 12 (*p* < 0.001 for both).

The WCO31 group showed a significantly higher height increment at 24 weeks than the placebo group (3.12 ± 0.92 cm vs. 2.38 ± 0.82 cm; *p* < 0.001). This persisted even after adjusting for compliance (*p* < 0.001; Effect size = 0.74; 95% CIs, 0.45–1.02). Both groups demonstrated significant within-group growth across visits (*p* < 0.001 for both). In the intention-to-treat (ITT) analysis, where missing data were addressed using multiple imputation, the WCO31 group showed a numerically greater height increment than the placebo group at 24 weeks, with an effect size (0.77 cm) consistent with the PP analysis. However, the difference in the ITT population did not reach statistical significance (95% CIs, −1.63–3.17; *p* = 0.528; Table S3). This divergence between the PP and ITT results likely stems from the inclusion of participants with lower treatment compliance in the ITT population, which may have led to a dilution of the detectable treatment effect. Furthermore, the inherent variability introduced by the multiple imputation process for missing values may have further reduced the statistical power in the ITT analysis compared to the PP analysis.

#### 3.4.3. Secondary Efficacy Analysis

Secondary efficacy analysis variables were assessed at baseline and week 24.

##### Growth Rate, Growth Rate SDS and Height SDS

A comparison of the growth rate changes from baseline to week 24 revealed a significant between-group difference (*p* < 0.001). Specifically, the WCO31 group exhibited a markedly higher increase (1.63 ± 0.95) than the placebo group (1.07 ± 0.79). After adjusting for compliance and baseline values, the treatment effect remained robust, with a standardized effect size (0.56 [95% CIs: 0.28–0.83]).

The change in growth rate SDS differed significantly between the groups (*p* < 0.001); the WCO31 group exhibited a markedly higher increase of 4.06 ± 3.04 compared with 2.60 ± 1.97 in the placebo group. The significant between-group difference persisted even after adjusting for both compliance and baseline growth rate SDS (*p* < 0.001), demonstrating a large effect size (1.46 [95% CIs: 0.76–2.16]).

Furthermore, the WCO31 group demonstrated a significantly greater improvement in height SDS than the placebo group (*p* < 0.001). Results showed a positive increment of 0.06 ± 0.13 in the WCO31 group, in contrast to a slight decline of −0.03 ± 0.11 in the placebo group. This improvement in height SDS was associated with a small effect size (0.08 [95% CIs: 0.05–0.12]) after adjusting for covariates.

Significant between-group differences were observed in the growth rate, growth rate SDS, and height SDS across the study visits (all *p* < 0.001). These significant discrepancies remained consistent even after adjusting for compliance and baseline growth rate (all *p* < 0.001, Table 7).

Subgroup analysis based on baseline growth status.

To evaluate whether the treatment effect was influenced by baseline growth status, a subgroup analysis was conducted by stratifying participants based on the median baseline growth rate SDS (−2.188). The results demonstrated a consistent and significant superiority of WCO31 over the placebo in both subgroups. Specifically, in the lower baseline subgroup (SDS ≤ −2.188), WCO31 showed a significantly greater improvement than placebo at week 24 (difference = 2.01, *p* < 0.001). Notably, even in the upper baseline subgroup (SDS > −2.188) with relatively higher initial values, WCO31 maintained a significant improvement (difference = 0.61, *p* = 0.019). These findings indicate that the efficacy of WCO31 is robust and independent of baseline growth profiles (Table 8).

##### Growth-Regulating Factors, Bone-Related Indicators, and Growth-Related Z-Score

Regarding growth-regulating factors, no significant between-group differences in the changes in IGF-1, IGFBP3, or the IGF-1/IGFBP3 ratio from baseline to week 24 (*p* > 0.05) were observed. No significant between-group differences were observed in bone-related biomarkers, including bone age or osteocalcin levels, at the end of the study period (*p* > 0.05). In contrast, a significant between-group difference was observed in the mean change in HAZ (*p* < 0.001). Although the WCO31 group demonstrated an improvement (0.10 ± 0.22), the placebo group experienced a slight reduction in scores (−0.04 ± 0.19). Significant between-group differences persisted in the compliance-adjusted analyses (*p* < 0.001; Effect size = 0.17; 95% CIs: −0.07–0.42). Conversely, there were no significant differences in changes in WHZ, another growth-related Z-score (*p* > 0.05; Table 9).

### 3.5. Safety Analysis

A safety analysis was conducted on 160 participants (safety set) who participated in the study and had at least one sample taken.

#### 3.5.1. AEs

Overall, AEs were reported in 21 of the 160 participants, with 12 cases occurring in the WCO31 group and nine in the placebo group. The incidence of AEs did not differ significantly between the two study groups (*p* > 0.05). Mild AEs, such as common cold, cervical sprain/strain, allergic rhinitis, otitis media, bronchitis, influenza A, and fever, occurred; however, these events were judged to be unrelated to the consumption of the sample.

#### 3.5.2. Laboratory Tests

Laboratory tests, including complete blood count, biochemistry tests, urinalysis, and sex hormone tests, showed no significant between-group variations from baseline to week 24 (*p* > 0.05) (Table 10).

#### 3.5.3. Vital Signs

Vital signs were evaluated at baseline (screening) and at week 24 (visit 2). No significant between-group differences were identified for any of these parameters (*p* > 0.05; Table 11).

## 4. Discussion

In this study, we investigated the efficacy and safety of WCO31 (*A. fistulosum* L. root and *A. sativa* L. mixtures) on height growth in children. Analysis of the 24-week study data revealed significant variations in sample intake compliance and baseline growth rate SDSs (*p* = 0.036 and *p* < 0.001). The primary endpoint (height increment) was significantly greater in the WCO31 group than in the placebo group, with a net difference of 0.74 cm over 24 weeks (*p* < 0.001). This result compares favorably with the findings of previous clinical trials of natural extracts. For instance, a study on the Astragalus extract mixture HT042 reported a 0.45 cm increase relative to placebo over the same period [22]. While a 0.74 cm difference may appear modest in a clinical context compared to hormonal therapies, it is noteworthy as a non-pharmacological intervention. Furthermore, this extract has been officially recognized by the MFDS as a functional ingredient for health functional foods aimed at supporting physical growth [23]. While recombinant human growth hormone therapy is a primary medical intervention for growth disorders, it presents several therapeutic challenges. Long-term GH therapy is associated with potential risks, including insulin resistance, increased levels of IGF-1 that necessitate careful monitoring for malignancy, and the high cost and invasive nature of daily injections [24,25]. Moreover, the ethical debate surrounding the “medicalization” of height in children with idiopathic short stature remains a concern in clinical practice [26]. Given these medical and practical hurdles, WCO31 serves as a safe and accessible complementary option to support longitudinal growth in children. Regarding the secondary efficacy variables, the WCO31 group demonstrated superior improvements across all parameters, including changes in growth rate, growth rate SDS, height SDS, and HAZ, compared with the placebo group (all *p* < 0.001). A previous in vivo study showed that a mixture of green onion and oat water-soluble extracts administered for 4 weeks increased serum GH and osteoprotegerin concentrations and increased femur and tibia length, similar to a positive control group administered human GH [20]. GH and IGF-1 regulate bone homeostasis and play a key role in longitudinal bone growth [27]. Osteoprotegerin protects the skeleton by binding to RANKL in RANK signaling, preventing RANKL from binding to its receptor RANK, thereby preventing bone resorption by osteoclasts [28]. The significant increase in height in the WCO31 group in this clinical trial is consistent with findings from a previous in vivo study [20].

Ryuk et al. [14] found that the *A. fistulosum* bulb extracts increased the growth plate height, BMC, and density of the femur and tibia in calcium- and vitamin D-deficient C57BL/6 mice. Additionally, water and 30% ethanol extracts of *A. fistulosum* bulbs increase ALP activity in MC3T3-E1 and MG63 cells [14]. ALP is a major marker of osteoblast differentiation [29]. Collagen facilitates osteoblast differentiation by upregulating bone formation-associated genes and modulating ALP activity, while attenuating the expression of the pro-inflammatory cytokine TNF-α [30]. ALP also acts as a biomineralization enzyme; it increases inorganic phosphate concentration and accumulates calcium within matrix vesicles, contributing to bone formation through extracellular matrix mineralization [31]. Therefore, *A. fistulosum* bulb extract increases ALP activity and promotes osteoblast differentiation and bone formation, thereby increasing bone density and length. Ko et al. demonstrated that *A. fistulosum* root water extracts promote longitudinal femur and tibia growth by expanding the proliferative and hypertrophic zone of the growth plate through activation of IGF-1 and TGF-β signaling [32]. IGF-1 and insulin bind to their respective receptors and promote IRS-2 phosphorylation and expression. IRS-2 plays a crucial role in bone formation by inhibiting osteoclast formation and RANKL expression in osteoblasts [33]. In our study, although IGF-1 levels and IGF-1/IGFBP-3 ratio showed significant within-group increases after the intervention (*p* = 0.041 and *p* = 0.025, respectively), no significant differences were observed between the WCO31 and placebo groups. This discrepancy can be interpreted through the complex interplay between systemic and local IGF-1 actions. Unlike animal models, where growth deficiency is often artificially induced to observe dramatic hormonal shifts, healthy humans possess homeostatic mechanisms that may limit substantial changes in systemic IGF-1 concentrations [34]. Furthermore, as highlighted by Yakar et al. [35], longitudinal bone growth is significantly driven by local paracrine/autocrine IGF-1 signaling within the growth plate, which can operate independently of circulating levels. This leads us to hypothesize that the growth-promoting effect of WCO31 could be mediated by enhancing the localized activity of chondrocytes or increasing the sensitivity of the growth plate to available growth factors, rather than inducing a major shift in the systemic GH-IGF-1 axis. Therefore, while these biomarker trends are consistent with the direction of previous preclinical findings, the precise role of the systemic IGF-1 signaling pathway in the efficacy of WCO31 requires further elucidation. Additional research is warranted to clarify the specific molecular mechanisms involving localized IGF-1 and its associated signaling molecules in human longitudinal bone growth.

The growth-promoting efficacy of WCO31 is further supported by the phytochemical properties of its constituents. *A. sativa* L. is particularly rich in β-glucan and essential minerals, including magnesium, phosphorus, and calcium, which are fundamental components for bone matrix mineralization and the maintenance of bone density [36,37,38]. While research on the direct effects of oat-derived β-glucan on human bone metabolism is still evolving, a previous study demonstrated that supplementation with oat β-glucan increased trabecular bone thickness in growing Wistar rats [39]. Furthermore, *A. fistulosum* contains bioactive flavonoids such as quercetin, which has been shown to enhance osteoblast differentiation and bone formation by promoting the expression of bone-related genes [40]. Additionally, previous studies have demonstrated that *A. fistulosum* extracts can increase ALP activity, a key marker for bone mineralization [14]. These findings suggest that the functional components in oats, combined with the ALP-stimulating effects of *A. fistulosum* root extracts, provide a supportive nutritional environment for longitudinal bone growth and structural development.

This study has several limitations. First, the 24-week intervention period may have been insufficient to fully evaluate the effects of WCO31, although the effects observed at 24 weeks were more evident than those at 12 weeks. Second, although changes in IGF-1 levels were observed in both the WCO31 and control groups, these differences were not statistically significant. Therefore, further studies elucidating the physiological mechanisms underlying bone growth are warranted. Third, although diet, outdoor activity, and sleep habits were monitored via surveys, these variables were not strictly controlled or objectively measured. Relying on self-reported data may introduce reporting bias and potential confounding effects on growth outcomes. Finally, the relatively small sample size and the inclusion of exclusively Korean children from specific centers may limit the external validity and generalizability of the findings to other ethnic groups or geographical regions. Future multi-center studies involving diverse ethnic cohorts are required to confirm the broader clinical efficacy.

Despite these limitations, this study has several noteworthy strengths. First, it was conducted as a randomized, double-blind, placebo-controlled, multi-center trial, providing a high level of clinical evidence. Second, the study design and clinical protocols were strictly aligned with the MFDS guidelines for the functional evaluation of health functional foods. Furthermore, the study was conducted after obtaining Institutional Review Board (IRB) approval from each participating medical center, ensuring high methodological rigor and ethical standards. Third, the 24-week safety profile of WCO31 was confirmed with no observable adverse effects, supporting its potential as a safe long-term nutritional intervention for children.

## 5. Conclusions

In this study, we provide clinical evidence that daily administration of WCO31 promotes growth in children exhibiting delayed development. Our results substantiate the efficacy observed in earlier preclinical studies and further validate the biological impact of this material composition on stature. The safety of the intervention was also established. This study provides a scientific basis for utilizing botanical ingredients in the formulation of growth-supporting health supplements, offering a potentially superior safety-to-efficacy ratio compared to existing options. To build upon these findings, future studies should focus on long-term follow-up to assess the sustained impact on final adult height and investigate the underlying mechanisms, such as the modulation of specific growth factors. Additionally, large-scale multi-center trials involving diverse ethnic populations are warranted to confirm the broader generalizability and clinical utility of WCO31.

## Figures and Tables

**Table 1:** Eligibility criteria for study participants.

Category	Criteria
Inclusion criteria	Male and female children aged 6–8 years at the point of initial screening. Height between the 3rd and 50th percentile (2017 Korean National Growth charts). Absence of secondary sexual characteristics.
Exclusion criteria	History of hormone replacement therapy or phosphonate, calcitonin, or growth hormone use within 6 months of the initial screening test. Endocrine disorders causing growth retardation (e.g., GH deficiency, hypothyroidism, type 1 diabetes). Chromosomal or genetic abnormalities or other syndromes. Rapid weight change (> 5%) within the previous year. Severe anemia (0.5–6 years: Hb ≤ 11 g/dL, 6–14 years: Hb < 12 g/dL). Premature infants (<37 weeks of gestation) and infants with low birth weight (<2.5 kg) excluding those who had shown normal growth and development after birth. Underweight (weight-for-age < 5th percentile according to WHO standards). Precocious puberty or onset of menstruation. Thyroid hormone levels (TSH, T3, fT4) outside the institutional reference range (subject to the principal investigator’s discretion based on medical assessment). Use of ADHD medication. Clinically relevant systemic diseases requiring medical treatment (e.g., immunological, endocrine, respiratory, cardiovascular, hematological, inflammatory, oncological, musculoskeletal, neuropsychiatric, gastrointestinal, renal, hepatobiliary, or urological disorders). Use of growth-related medications or health functional foods within the preceding 3 months. Administration of antipsychotic drugs within the preceding 3 months. Enrollment in other clinical trials within the preceding 3 months. Abnormal laboratory findings: AST or ALT > 3 times upper limit of normal or serum creatinine > 2.0 mg/dL. Any other medical considerations or clinical laboratory findings judged ineligible by the principal investigator.

ADHD, attention-deficit hyperactivity disorder; ALT, alanine aminotransferase; AST, aspartate aminotransferase; fT4, free thyroxine; GH, growth hormone; Hb, hemoglobin concentration; T3, triiodothyronine; TSH, thyroid-stimulating hormone.

**Table 2:** Demographic and clinical characteristics of the study population at baseline.

Variables	WCO31 Group (*n* = 80)	Placebo Group (*n* = 80)	Total (*n* = 160)	*p* Value ^(1)^
Age (years)	6.69 ± 0.76	6.93 ± 0.79	6.81 ± 0.78	0.054
Height (cm)	118.90 ± 5.26	120.29 ± 5.41	119.59 ± 5.36	0.101
Weight (kg)	22.20 ± 3.67	23.17 ± 4.62	22.69 ± 4.19	0.142
BMI (kg/m^2^)	15.64 ± 1.86	15.90 ± 2.15	15.77 ± 2.01	0.415
SBP (mmHg)	108.63 ± 12.45	108.38 ± 10.80	108.50 ± 11.62	0.892
DBP (mmHg)	70.85 ± 11.58	71.36 ± 10.65	71.11 ± 11.09	0.771
Pulse (beats/min)	87.41 ± 9.10	89.13 ± 8.55	88.27 ± 8.84	0.222
Standard growth chart	28.99 ± 14.59	28.44 ± 14.90	28.71 ± 14.70	0.813
Birth length (cm)	50.27 ± 2.29	49.98 ± 2.42	50.13 ± 2.35	0.431
Birth weight (kg)	3.14 ± 0.39	3.17 ± 0.40	3.15 ± 0.39	0.576
Outdoor activity time (h/day)	0.84 ± 0.86	0.81 ± 0.83	0.82 ± 0.84	0.816

Values are presented as mean ± SD. ^(1)^ Statistical comparisons between groups were performed using independent *t*-tests. BMI, body mass index; DBP, diastolic blood pressure; SBP, systolic blood pressure; SD, standard deviation.

**Table 3:** Efficacy analysis at baseline.

Variables	WCO31 Group (*n* = 73)	Placebo Group (*n* = 77)	Total (*n* = 150)	*p* Value ^(1)^
Primary efficacy analysis				
Height (cm)	119.48 ± 5.14	120.65 ± 5.55	120.08 ± 5.37	0.186
Secondary efficacy analysis				
Growth rate	0.29 ± 0.31	0.36 ± 0.46	0.33 ± 0.40	0.245
Growth rate SDS	−3.07 ± 1.36	−2.03 ± 1.21	−2.54 ± 1.38	<0.001 ***
Height SDS	−0.40 ± 0.31	−0.41 ± 0.33	−0.41 ± 0.32	0.751
Growth regulation indicators				
IGF-1 (ng/mL)	148.25 ± 48.85	146.70 ± 47.92	147.45 ± 48.22	0.846
IGFBP-3 (ng/mL)	3845.70 ± 641.52	3802.06 ± 709.39	3823.30 ± 675.30	0.694
IGF-1/IGFBP-3 ratio	0.14 ± 0.03	0.14 ± 0.03	0.14 ± 0.03	0.951
Growth hormone (ng/mL)	2.16 ± 3.14	2.58 ± 3.18	2.38 ± 3.16	0.422
Bone-related indicators				
Bone age (months)	81.03 ± 14.37	81.47 ± 12.32	81.25 ± 13.31	0.840
Osteocalcin (ng/mL)	71.01 ± 14.74	71.70 ± 17.76	71.37 ± 16.31	0.796
Growth-related Z-score				
WHZ	−0.13 ± 1.20	−0.14 ± 1.20	−0.13 ± 1.20	0.935
HAZ	−0.57 ± 0.51	−0.61 ± 0.54	−0.59 ± 0.52	0.654

Values are presented as mean ± SD. ^(1)^ Statistical comparisons between groups were performed using independent *t*-tests. *** *p* < 0.001. HAZ, height-for-age Z-score; IGF-1, insulin-like growth factor-1; IGFBP-3, insulin-like growth factor binding protein-3; SD, standard deviation; SDS, standard deviation score; WHZ, weight-for-height Z-score.

**Table 4:** Height changes at baseline and after 12 weeks.

Variable	WCO31 Group (*n* = 73)			Placebo Group (*n* = 77)			*p* Value ^(2)^	*p* Value ^(3)^	Effect Size ^(4)^ (95% CIs)
	Baseline	12 Weeks	*p* Value ^(1)^	Baseline	12 Weeks	*p* Value ^(1)^			
	Change Value			Change Value					
Height (cm)	119.48 ± 5.14	120.68 ± 5.11	<0.001 ***	120.65 ± 5.55	121.60 ± 5.61	<0.001 ***	0.032 *	0.032 *	0.25 (0.02, 0.47)
	1.20 ± 0.70			0.95 ± 0.69					

Values are presented as mean ± SD. ^(1)^ Paired *t*-tests were performed for within-group comparisons. ^(2)^ Independent *t*-tests were performed for between-group comparisons of the mean changes. ^(3)^ ANCOVA was performed to compare outcomes between the groups, adjusted for compliance. ^(4)^ Between-group effect sizes (estimated mean difference with 95% CIs) were estimated using LMMs adjusted for compliance. * *p* < 0.05, *** *p* < 0.001. ANCOVA, analysis of covariance; CI, confidence interval; LMM, linear mixed model; SD, standard deviation.

**Table 5:** Height changes at baseline and after 24 weeks.

Variable	WCO31 Group (*n* = 73)			Placebo Group (*n* = 77)			*p* Value ^(2)^	*p* Value ^(3)^	Effect Size ^(4)^ (95% CIs)
	Baseline	24 Weeks	*p* Value ^(1)^	Baseline	24 Weeks	*p* Value ^(1)^			
	Change Value			Change Value					
Height (cm)	119.48 ± 5.14	122.60 ± 5.30	<0.001 ***	120.65 ± 5.55	123.03 ± 5.61	<0.001 ***	<0.001 ***	<0.001 ***	0.74 (0.45, 1.02)
	3.12 ± 0.92			2.38 ± 0.82					

Values are presented as mean ± SD. ^(1)^ Paired *t*-tests were performed for within-group comparisons. ^(2)^ Independent *t*-tests were performed for between-group comparisons of the mean changes. ^(3)^ ANCOVA was performed to compare outcomes between the groups, adjusted for compliance. ^(4)^ Between-group effect sizes (estimated mean difference with 95% CIs) were estimated using LMMs adjusted for compliance. *** *p* < 0.001. ANCOVA, analysis of covariance; CI, confidence interval; LMM, linear mixed model; SD, standard deviation.

**Table 6:** Height changes by visit over 24 weeks.

Variable	WCO31 Group (*n* = 73)				Placebo Group (*n* = 77)				*p* Value ^(1)^	*p* Value ^(2)^	Effect Size ^(3)^ (95% CIs)
	Base Line	12 Weeks	24 Weeks	*p* Value ^(1)^	Base Line	12 Weeks	24 Weeks	*p* Value ^(1)^			
Height (cm)	119.48 ± 5.14 ^c^	120.68 ± 5.11 ^b^	122.60 ± 5.30 ^a^	<0.001 ***	120.65 ± 5.55 ^b^	121.60 ± 5.61 ^b^	123.03 ± 5.61 ^a^	<0.001 ***	<0.001 ***	<0.001 ***	0.74 (0.45, 1.02)

Values are presented as mean ± SD. ^(1)^ Repeated-measures data were analyzed using linear mixed models with Bonferroni post hoc tests applied (a > b > c). ^(2)^ ANCOVA was performed to compare the groups, adjusted for compliance. ^(3)^ Between-group effect sizes (estimated mean difference with 95% CIs) were estimated using LMMs adjusted for compliance. *** *p* < 0.001. ANCOVA, analysis of covariance; CI, confidence interval; LMM, linear mixed model; SD, standard deviation.

**Table 7:** Changes in growth rate, growth rate SDS, and height SDS at baseline and after 24 weeks.

Variables	WCO31 Group (*n* = 73)			Placebo Group (*n* = 77)			*p* Value ^(2)^	*p* Value ^(3)^	Effect Size ^(5)^ (95% CIs)
	Baseline	24 Weeks	*p* Value ^(1)^	Baseline	24 Weeks	*p* Value ^(1)^			
	Change Value			Change Value					
Growth rate	0.29 ± 0.31	1.92 ± 0.83	<0.001 ***	0.36 ± 0.46	1.43 ± 0.63	<0.001 ***	<0.001 ***	<0.001 ***	0.56 (0.28, 0.83)
	1.63 ± 0.95			1.07 ± 0.79					
Growth rate SDS	−3.07 ± 1.36	0.99 ± 2.18	<0.001 ***	−2.03 ± 1.21	0.57 ± 1.47	<0.001 ***	<0.001 ***	<0.001 ***^,(4)^	1.46 (0.76, 2.16)
	4.06 ± 3.04			2.06 ± 1.97					
Height SDS	−0.40 ± 0.31	−0.34 ± 0.34	<0.001 ***	−0.41 ± 0.33	−0.44 ± 0.36	0.0413 *	<0.001 ***	<0.001 ***	0.08 (0.05, 0.12)
	0.06 ± 0.13			−0.03 ± 0.11					

Values are presented as mean ± SD. ^(1)^ Paired *t*-tests were performed for within-group comparisons. ^(2)^ Independent *t*-tests were performed for between-group comparisons of the mean changes. ^(3)^ ANCOVA was performed to compare the groups, adjusted for compliance. ^(4)^ ANCOVA was performed to compare the groups, adjusted for compliance and baseline. ^(5)^ Between-group effect sizes (estimated mean difference with 95% CIs) were estimated using LMMs adjusted for compliance. * *p* < 0.05, *** *p* < 0.001. ANCOVA, analysis of covariance; CI, confidence interval; LMM, linear mixed model; SD, standard deviation.

**Table 8:** Subgroup analysis of growth rate SDS according to baseline levels (24 weeks).

Subgroup (Baseline Growth Rate SDS)	Group	Baseline	Week 24	Difference ^(1)^ (T-P)	*p* Value ^(2)^
Lower group (≤−2.188)	WCO31 (T)	−3.26 ± 0.24	1.86 ± 0.24	2.01	<0.001 ***
	Placebo (P)	−2.74 ± 0.28	−0.15 ± 0.28		
Upper group (≥−2.188)	WCO31 (T)	−1.56 ± 0.20	0.91 ± 0.20	0.61	0.019 *
	Placebo (P)	−1.38 ± 0.16	0.30 ± 0.16		

Values are presented as LS means ± SE, estimated using LMMs adjusted for baseline growth rate SDS and treatment compliance. Participants were stratified into two subgroups based on the median baseline growth rate SDS (Median = −2.188). ^(1)^ Difference represents the LS mean of the WCO31 group minus the LS mean of the placebo group. ^(2)^ LMMs were performed to compare the WCO31 and placebo group at week 24 within each subgroup. * *p* < 0.05, *** *p* < 0.001. LMM, linear mixed model; LS means, least squares means; SDS, standard deviation score; SE, standard error.

**Table 9:** Changes in growth-regulating factors, bone-related indicators, and growth-related Z-score at baseline and after 24 weeks.

Variables	WCO31 Group (*n* = 73)			Placebo Group (*n* = 77)			*p* Value ^(2)^	*p* Value ^(3)^	Effect Size ^(4)^ (95% CIs)
	Baseline	24 Weeks	*p* Value ^(1)^	Baseline	24 Weeks	*p* Value ^(1)^			
	Change Value			Change Value					
IGF-1 (ng/mL)	148.25 ± 48.85	155.11 ± 49.11	0.041 *	146.70 ± 47.92	151.33 ± 44.86	0.201	0.648	0.648	0.97 (−19.89, 21.84)
	6.86 ± 28.24			4.63 ± 31.43					
IGFBP-3 (ng/mL)	3845.70 ± 641.52	3858.74 ± 682.93	0.775	3802.06 ± 709.39	3900.90 ± 596.73	0.113	0.265	0.270	−78.30 (−367.09, 210.48)
	13.04 ± 388.68			98.83 ± 541.53					
IGF-1/ IGFBP-3 ratio	0.14 ± 0.03	0.15 ± 0.03	0.025 *	0.14 ± 0.03	0.14 ± 0.03	0.557	0.199	0.199	0.00 (−0.01, 0.02)
	0.03 ± 0.02			0.00 ± 0.02					
Growth hormone (ng/mL)	2.16 ± 3.14	1.47 ± 2.91	0.136	2.58 ± 3.18	1.33 ± 2.53	0.003 **	0.365	0.365	0.05 (−1.25, 1.35)
	−0.69 ± 3.94			−1.25 ± 3.57					
Bone age (months)	81.03 ± 14.37	87.60 ± 13.05	<0.001 ***	81.47 ± 12.32	87.45 ± 12.72	<0.001 ***	0.482	0.482	0.14 (−5.69, 5.97)
	6.58 ± 5.55			5.99 ± 4.66					
Osteocalcin (ng/mL)	71.01 ± 14.74	73.66 ± 15.83	0.147	71.70 ± 17.76	74.78 ± 17.68	0.127	0.875	0.875	−1.57 (−8.90, 5.75)
	2.65 ± 15.43			3.07 ± 17.47					
WHZ	−0.13 ± 1.20	0.03 ± 1.07	0.019 *	−0.14 ± 1.20	0.05 ± 1.19	0.001 **	0.661	0.661	−0.05 (−0.57, 0.47)
	0.15 ± 0.55			0.19 ± 0.50					
HAZ	−0.57 ± 0.51	−0.47 ± 0.55	<0.001 ***	−0.61 ± 0.54	−0.65 ± 0.59	0.067	<0.001 ***	<0.001 ***	0.17 (−0.07, 0.42)
	0.10 ± 0.22			−0.04 ± 0.19					

Values are presented as mean ± SD. ^(1)^ Paired *t*-tests were performed for within-group comparisons. ^(2)^ Independent *t*-tests were performed for between-group comparisons of the mean changes. ^(3)^ ANCOVA was performed to compare the groups, adjusted for compliance. ^(4)^ Between-group effect sizes (estimated mean difference with 95% CIs) were estimated using LMMs adjusted for compliance. * *p* < 0.05, ** *p* < 0.01, *** *p* < 0.001. ANCOVA, analysis of covariance; CI, confidence interval; HAZ, height-for-age Z-score; IGF-1, insulin-like growth factor-1; IGFBP-3, insulin-like growth factor binding protein-3; LMM, linear mixed model; SD, standard deviation; WHZ, weight-for-height Z-score.

**Table 10:** Changes in laboratory tests at baseline and after 24 weeks.

	WCO31 Group (*n* = 80)			Placebo Group (*n* = 80)			*p* Value ^(2)^
	Baseline	24 Weeks	*p* Value ^(1)^	Baseline	24 Weeks	*p* Value ^(1)^	
	Change Value			Change Value			
Complete blood count							
White blood cell	6.30 ± 0.24	6.80 ± 0.24	0.137	6.69 ± 0.24	6.68 ± 0.24	0.716	0.285
	0.50 ± 0.34			−0.01 ± 0.33			
Red blood cell	4.70 ± 0.03	4.70 ± 0.03	0.993	4.70 ± 0.03	4.72 ± 0.03	0.556	0.682
	0.00 ± 0.05			0.03 ± 0.05			
Hemoglobin	12.95 ± 0.07	12.98 ± 0.07	0.814	13.07 ± 0.07	13.10 ± 0.07	0.792	0.985
	0.02 ± 0.10			0.02 ± 0.10			
Hematocrit	38.40 ± 0.30	38.40 ± 0.30	0.989	38.62 ± 0.30	39.07 ± 0.30	0.289	0.449
	0.01 ± 0.43			0.45 ± 0.42			
Platelet	313.09 ± 7.40	302.94 ± 7.54	0.337	300.15 ± 7.40	304.09 ± 7.43	0.707	0.344
	−10.15 ± 10.57			3.94 ± 10.49			
Biochemistry tests (U/L)							
Alkaline phosphatase	237.74 ± 8.17	241.23 ± 8.35	0.701	262.26 ± 8.17	256.34 ± 8.24	0.610	0.527
	4.49 ± 11.69			−5.92 ± 11.60			
Aspartate aminotransferase	30.76 ± 0.70	29.97 ± 0.71	0.430	31.33 ± 0.70	29.60 ± 0.70	0.080	0.506
	−0.79 ± 0.99			−1.73 ± 0.99			
Alanine aminotransferase	15.51 ± 1.03	15.15 ± 1.04	0.805	17.59 ± 1.03	15.64 ± 1.04	0.184	0.445
	−0.36 ± 1.46			−1.94 ± 1.46			
Total bilirubin	0.62 ± 0.03	0.64 ± 0.03	0.574	0.59 ± 0.03	0.63 ± 0.03	0.288	0.726
	0.02 ± 0.04			0.04 ± 0.04			
Total protein	7.20 ± 0.04	7.14 ± 0.04	0.338	7.18 ± 0.04	7.09 ± 0.04	0.105	0.642
	−0.05 ± 0.06			−0.09 ± 0.06			
Albumin	4.53 ± 0.03	4.54 ± 0.03	0.917	4.54 ± 0.03	4.53 ± 0.03	0.793	0.796
	0.00 ± 0.04			−0.01 ± 0.04			
Gamma-GT	12.56 ± 0.34	11.45 ± 0.35	0.024 *	12.41 ± 0.34	11.42 ± 0.34	0.041 *	0.866
	−1.11 ± 0.49			−0.99 ± 0.49			
Blood urea nitrogen	12.20 ± 0.30	12.50 ± 0.31	0.481	11.93 ± 0.30	11.99 ± 0.30	0.890	0.688
	0.30 ± 0.43			0.06 ± 0.43			
Creatinine	0.52 ± 0.01	0.45 ± 0.01	<0.001 ***	0.53 ± 0.01	0.45 ± 0.01	<0.001 ***	0.886
	−0.08 ± 0.01			−0.08 ± 0.01			
Total cholesterol	177.54 ± 3.44	178.68 ± 3.54	0.817	172.26 ± 3.44	176.57 ± 3.47	0.378	0.648
	1.14 ± 4.94			4.31 ± 4.88			
Triglyceride	67.79 ± 3.68	65.51 ± 3.79	0.667	68.24 ± 3.68	71.51 ± 3.71	0.531	0.456
	−2.28 ± 5.29			3.27 ± 5.23			
LDL cholesterol	102.53 ± 3.04	99.26 ± 3.11	0.452	102.27 ± 3.04	101.04 ± 3.06	0.776	0.738
	−3.28 ± 4.35			−1.23 ± 4.32			
HDL cholesterol	63.40 ± 1.28	63.40 ± 1.31	0.957	60.95 ± 1.28	60.70 ± 1.30	0.890	0.892
	0.01 ± 1.83			−0.25 ± 1.82			
Glucose	92.10 ± 0.78	93.29 ± 0.79	0.283	92.54 ± 0.78	94.10 ± 0.78	0.158	0.815
	1.19 ± 1.11			1.55 ± 1.10			
Lactate dehydrogenase	234.53 ± 4.08	236.66 ± 4.15	0.713	243.20 ± 4.08	240.72 ± 4.10	0.668	0.574
	2.14 ± 5.82			−2.48 ± 5.78			
Creatine kinase	118.21 ± 4.48	124.12 ± 4.56	0.355	125.03 ± 4.48	125.79 ± 4.50	0.905	0.568
	5.91 ± 6.39			0.76 ± 6.36			
High sensitivity C-reactive protein	0.62 ± 0.20	1.00 ± 0.20	0.179	1.15 ± 0.20	0.93 ± 0.20	0.453	0.139
	0.38 ± 0.28			−0.21 ± 0.28			
Urinalysis							
Specific gravity	1.02 ± 0.00	1.02 ± 0.00	0.888	1.02 ± 0.00	1.02 ± 0.00	0.719	0.724
	−0.00 ± 0.00			0.00 ± 0.00			
pH	5.82 ± 0.09	5.75 ± 0.09	0.693	5.78 ± 0.09	5.78 ± 0.09	0.987	0.789
	−0.05 ± 0.12			0.00 ± 0.12			
Sex hormone test							
Estradiol 2 hormone	5.08 ± 0.39	5.34 ± 0.40	0.644	5.07 ± 0.39	6.09 ± 0.40	0.068	0.335
	0.26 ± 0.56			1.02 ± 0.56			
Luteinizing hormone	0.32 ± 0.04	0.31 ± 0.04	0.762	0.32 ± 0.04	0.39 ± 0.04	0.190	0.254
	−0.02 ± 0.05			0.07 ± 0.05			
Total testosterone	0.03 ± 0.00	0.03 ± 0.00	0.973	0.03 ± 0.00	0.02 ± 0.00	0.078	0.223
	0.00 ± 0.00			−0.00 ± 0.00			

Values are presented as LS means ± SE. Statistical analysis was performed based on the Safety set. Missing data were handled by 20 iterations of MI using the FCS method. All results from the imputed datasets were pooled according to Rubin’s rules to derive final estimates and *p*-values. ^(1)^ Within-group mean changes from baseline were compared using LMMs. ^(2)^ Between-group differences in changes were evaluated by analyzing the group-by-visit interaction effect in the model. * *p* < 0.05, *** *p* < 0.001. FCS, fully conditional specification; Gamma-GT, gamma-glutamyl transferase; HDL-Cholesterol, high-density lipoprotein cholesterol; LDL-Cholesterol, low density lipoprotein cholesterol; LMM, linear mixed model; LS means, least squares means; MI, multiple imputation; SE, standard error.

**Table 11:** Changes in vital signs at baseline and after 24 weeks.

	WCO31 Group (*n* = 80)			Placebo Group (*n* = 80)			*p* Value ^(2)^
	Baseline	24 Weeks	*p* Value ^(1)^	Baseline	24 Weeks	*p* Value ^(1)^	
	Change Value			Change Value			
SBP (mmHg)	108.73 ± 1.07	106.54 ± 1.08	0.151	109.75 ± 1.07	108.29 ± 1.07	0.335	0.736
	−2.18 ± 1.52			−1.46 ± 1.51			
DBP (mmHg)	71.19 ± 1.11	70.79 ± 1.13	0.800	71.25 ± 1.11	71.17 ± 1.12	0.957	0.888
	−0.40 ± 1.58			−0.08 ± 1.58			
Pulse (times/minute)	88.68 ± 0.93	86.63 ± 0.94	0.121	88.41 ± 0.93	86.09 ± 0.93	0.078	0.884
	−2.04 ± 1.32			−2.31 ± 1.32			

Values are presented as LS means ± SE. Statistical analysis was performed based on the Safety set. Missing data were handled by 20 iterations of MI using the FCS method. All results from the imputed datasets were pooled according to Rubin’s rules to derive final estimates and *p*-values. ^(1)^ Within-group mean changes from baseline were compared using LMMs. ^(2)^ Between-group differences in changes were evaluated by analyzing the group-by-visit interaction effect in the model. DBP, diastolic blood pressure; FCS, fully conditional specification; LMM, linear mixed model; LS means, least squares means; MI, multiple imputation; SBP, systolic blood pressure; SE, standard error.

## Data Availability

The data supporting the findings of this study can be obtained from the corresponding author upon request due to privacy.

## Supplementary Materials

The following supporting information can be downloaded at https://www.mdpi.com/article/10.3390/nu18091326/s1, Table S1: Baseline characteristics of efficacy parameters in the intention-to-treat (ITT) population. Table S2: Comparison of height and height changes from baseline to 12 weeks in the ITT population. Table S3: Comparison of height and height changes from baseline to 24 weeks in the ITT population. Table S4: Changes in height across 24 weeks of intervention based on the ITT population. Table S5: Changes in growth rate, growth rate SDS, and height SDS from baseline to 24 weeks in the ITT population. Table S6: Changes in growth-regulating factors, bone-related indicators, and growth-related Z-scores from baseline to 24 weeks in the ITT population.

## Abbreviations

The following abbreviations are used in this manuscript:

ADHD	Attention-deficit hyperactivity disorder
AEs	Adverse events
ALT	Alanine aminotransferase
AST	Aspartate aminotransferase
ANCOVA	Analysis of covariance
Gamma GT	Gamma-glutamyl transferase
GH	Growth hormone
HAZ	Height-for-age Z-score
IGF-1	Insulin-like growth factor-1
IGFBP-3	Insulin-like growth factor binding protein-3
ITT	Intention-to-treat
LMM	Linear mixed model
MFDS	Ministry of Food and Drug Safety
MI	Multiple imputation
PP	Per protocol
RM-ANOVA	Repeated measures-analysis of variance
SD	Standard deviation
SDS	Standard deviation score
SE	Standard error
WHZ	Weight-for-height Z-score
