# Supplementary material for: Effects of Allium fistulosum L. (Green Onion) Root and Avena sativa L. (Oat) Mixtures (WCO31) on the Height of Children: A Multi-Center, Randomized, Double-Blind, Placebo-Controlled Clinical Trial
Source: Nutrients. 2026 Apr 22;18(9):1326. doi: 10.3390/nu18091326 (PMC13164822; doi:10.3390/nu18091326)
Supplement: Supplementary file 1 [file nutrients-18-01326-s001.zip › nutrients-4242735-supplementary.pdf]

nutrients

MDPI

Supplementary Materials

Table S1. Baseline characteristics of efficacy parameters in the intention-to-treat (ITT) population.

Variables	WCO31 group (n = 80)	Placebo group (n = 80)	Total (n = 160)	p value (1)
**Primary efficacy analysis**				
Height (cm)	119.20 ± 5.24	120.60 ± 5.47	119.91 ± 5.39	0.088
**Secondary efficacy analysis**				
Growth rate	0.29 ± 0.31	0.35 ± 0.46	0.32 ± 0.39	0.303
Growth rate SDS	−2.66 ± 1.73	−1.93 ± 0.96	−2.30 ± 1.44	0.001**
Height SDS	−0.41 ± 0.32	−0.42 ± 0.33	−0.41 ± 0.33	0.822
**Growth regulation indicators**				
IGF-1 (ng/mL)	145.70 ± 48.27	145.50 ± 47.57	145.57 ± 47.77	0.978
IGFBP-3 (ng/mL)	3818.30 ± 622.30	3800.50 ± 699.00	3809.40 ± 659.73	0.865
IGF-1/IGFBP-3 ratio	0.14 ± 0.03	0.14 ± 0.03	0.14 ± 0.03	0.884
Growth hormone (ng/mL)	2.12 ± 3.04	2.68 ± 3.23	2.40 ± 3.14	0.264
**Bone-related indicators**				
Bone age (months)	80.95 ± 13.97	81.52 ± 12.62	81.24 ± 13.28	0.785
Osteocalcin (ng/mL)	70.01 ± 14.68	71.73 ± 17.59	70.87 ± 16.17	0.503
**Growth-related Z-score**				
WHZ	−0.16 ± 1.20	−0.16 ± 1.19	−0.16 ± 1.19	0.996
HAZ	−0.59 ± 0.53	−0.62 ± 0.54	−0.60 ± 0.53	0.721

Values are presented as mean ± SD. (1) Statistical comparisons between groups were performed using independent t-tests. ** $p < 0.01$. HAZ, height-for-age Z-score; IGF-1, insulin-like growth factor-1; IGFBP-3, insulin-like growth factor binding protein-3; SD, standard deviation; SDS, standard deviation score; WHZ, weight-for-height Z-score.

Table S2. Comparison of height and height changes from baseline to 12 weeks in the ITT population.

Variable	WCO31 group (*n* = 80) Baseline	WCO31 group (*n* = 80) 12 weeks	*p* value [(1)]	Placebo group (*n* = 80) Baseline	Placebo group (*n* = 80) 12 weeks	*p* value [(1)]	*p* value [(2)]	*p* value [(3)]	Effect size [(4)] (95%CIs)
	Change value			Change value					
Height (cm)	119.19 ± 0.61	120.31 ± 0.61	0.188	120.64 ± 0.61	121.60 ± 0.60	0.258	0.895	0.895	0.16 (−2.21, 2.52)
	1.21 ± 0.85			0.96 ± 0.85					

Values are presented as LS means ± SE. Statistical analysis was performed based on the ITT principle. Missing data were handled using MI to generate 20 complete datasets. All results from the imputed datasets were pooled according to Rubin's rules to derive final estimates and *p*-values. [(1)] Within-group mean changes from baseline were compared using LMMs. [(2)] Between-group differences in changes were evaluated by analyzing the group-by-visit interaction effect in the model. [(3)] The LMMs were further adjusted for treatment compliance as a covariate. [(4)] Between-group effect sizes (estimated mean difference with 95% CIs) were estimated using the MI-based LMMs adjusted for compliance. CI, confidence interval; ITT, intention-to-treat; LMM, linear mixed model; LS means, least squares means; MI, multiple imputation; SE, standard error.

Table S3. Comparison of height and height changes from baseline to 24 weeks in the ITT population.

Variable	WCO31 group (*n* = 80) Baseline	WCO31 group (*n* = 80) 24 weeks	*p* value [(1)]	Placebo group (*n* = 80) Baseline	Placebo group (*n* = 80) 24 weeks	*p* value [(1)]	*p* value [(2)]	*p* value [(3)]	Effect size [(4)] (95%CIs)
	Change value			Change value					
Height (cm)	119.19 ± 0.61	122.35 ± 0.62	< 0.001***	120.64 ± 0.61	123.03 ± 0.61	0.005**	0.528	0.528	0.77 (−1.63, 3.17)
	3.16 ± 0.87			2.39 ± 0.86					

Values are presented as LS means ± SE. Statistical analysis was performed based on the ITT principle. Missing data were handled using MI to generate 20 complete datasets. All results from the imputed datasets were pooled according to Rubin's rules to derive final estimates and p-values. [(1)] Within-group mean changes from baseline were compared using LMMs. [(2)] Between-group differences in changes were evaluated by analyzing the group-by-visit interaction effect in the model. [(3)] The LMMs were further adjusted for treatment compliance as a covariate. [(4)] Between-group effect sizes (estimated mean difference with 95% CIs) were estimated using the MI-based LMMs adjusted for compliance. ** $p < 0.01$. *** $p < 0.001$. CI, confidence interval; ITT, intention-to-treat; LMM, linear mixed model; LS means, least squares means; MI, multiple imputation; SE, standard error.

Table S4. Changes in height across 24 weeks of intervention based on the ITT population.

Variable	WCO31 group (*n* = 80) Base line	12 weeks	24 weeks	*p* value [(1)]	Placebo group (*n* = 80) Base line	12 weeks	24 weeks	*p* value [(1)]	*p* value [(1)]	*p* value [(2)]	Effect size [(3)] (95% CIs)
Height (cm)	119.19 ± 0.61	120.31 ± 0.61	122.35 ± 0.62	0.003**	120.64 ± 0.61	121.60 ± 0.60	123.03 ± 0.61	0.081	0.543	0.543	0.74 (−1.65, 3.13)

Values are presented as LS means ± SE. Statistical analysis was performed based on the ITT principle. Missing data were handled using MI to generate 20 complete datasets. All results from the imputed datasets were pooled according to Rubin's rules to derive final estimates and *p*-values. [(1)] Within- and

between-group comparisons were analyzed using LMMs for repeated-measures data, with Bonferroni correction for post-hoc pairwise comparisons. (2) Between-group differences in changes were evaluated by the group-by-visit interaction effect in LMMs adjusted for treatment compliance as a covariate. (3) Between-group effect sizes (estimated mean difference with 95% CIs) were estimated using the MI-based LMMs adjusted for compliance. $^{**}p < 0.01$. CI, confidence interval; ITT, intention-to-treat; LMM, linear mixed model; LS means, least squares means; MI, multiple imputation; SE, standard error.

Table S5. Changes in growth rate, growth rate SDS, and height SDS from baseline to 24 weeks in the ITT population.

Variables	WCO31 group (n = 80) Baseline	WCO31 group (n = 80) 24 weeks	WCO31 p value (1)	Placebo group (n = 80) Baseline	Placebo group (n = 80) 24 weeks	Placebo p value (1)	p value (2)	p value (3)	Effect size (5) (95% CIs)
	change value			change value					
Growth rate	0.29 ± 0.06	1.92 ± 0.07	< 0.001***	0.35 ± 0.06	1.42 ± 0.07	< 0.001***	< 0.001***	< 0.001***	0.56 (0.31, 0.82)
	1.63 ± 0.09			1.07±0.09					
Growth rate SDS	−3.08 ± 0.18	0.99 ± 0.18	< 0.001***	−2.06 ± 0.18	0.52 ± 0.18	< 0.001***	< 0.001***	< 0.001*** (4)	1.49 (0.81, 2.17)
	4.08 ± 0.25			2.59±0.25					
Height SDS	−0.41 ± 0.04	−0.36 ± 0.04	0.410	−0.42 ± 0.04	−0.44 ± 0.04	0.652	0.366	0.366	0.07 (−0.08, 0.22)
	0.05 ± 0.05			−0.02 ± 0.05					

Values are presented as LS means ± SE. Statistical analysis was performed based on the ITT principle. Missing data were handled using MI to generate 20 complete datasets. All results from the imputed datasets were pooled according to Rubin's rules to derive final estimates and p-values. (1) Within-group mean changes from baseline were compared using LMMs. (2) Between-group differences in changes were evaluated by analyzing the group-by-visit interaction effect in the model. (3) The LMMs were further adjusted for treatment compliance as a covariate. (4) An additional LMMs were applied adjusting for both treatment compliance and baseline values as covariates. (5) Between-group effect sizes (estimated mean difference with 95% CIs) were estimated using the MI-based LMMs adjusted for compliance. $^{***}p < 0.001$. CI, confidence interval; ITT, intention-to-treat; LMM, linear mixed model; LS means, least squares means; MI, multiple imputation; SDS, standard deviation score; SE, standard error.

Table S6. Changes in growth-regulating factors, bone-related indicators, and growth-related Z-scores from baseline to 24 weeks in the ITT population.

Varia-bles	WCO31 group (n = 80) Baseline	WCO31 group (n = 80) 24 weeks	p value (1)	Placebo group (n = 80) Baseline	Placebo group (n = 80) 24 weeks	p value (1)	p value (2)	p value (3)	Effect size (4) (95% CIs)
	change value			change value					
IGF-1 (ng/mL)	145.68 ± 5.27	153.94 ± 5.31	0.270	145.47 ± 5.27	151.31 ± 5.31	0.443	0.812	0.811	2.52 (−18.10, 23.14)
	8.26 ± 7.48			5.74 ± 7.48					
IGFBP-3 (ng/mL)	3818.30 ± 72.29	3856.46 ± 72.90	0.739	3800.50 ± 72.29	3907.19 ± 72.78	0.298	0.617	0.617	−72.52 (−356.35, 211.31)
	34.16 ± 102.69			106.69 ± 102.60					
IGF-1/	0.14 ± 0.00	0.15 ± 0.00	0.159	0.14 ± 0.00	0.14 ± 0.00	0.641	0.505	0.503	0.00

IGFBP-3 ratio	0.01 ± 0.00			0.00 ± 0.01					(−0.01, 0.02)
Growth hormone (ng/mL)	2.12 ± 0.34	1.52±0.35	0.223	2.68 ± 0.34	1.51 ± 0.35	0.017*	0.416	0.416	0.56
	−0.60 ± 0.49			−1.16 ± 0.49					(−0.79, 1.91)
Bone age (months)	80.95 ± 1.46	87.32 ± 1.49	0.002**	81.53 ± 1.46	87.77 ± 1.47	0.003**	0.965	0.965	0.13
	6.37 ± 2.09			6.24 ± 2.08					(−5.64, 5.90)
Osteo-calcin (ng/mL)	70.01 ± 1.83	73.79 ± 1.86	0.148	71.73 ± 1.83	74.56 ± 1.84	0.277	0.797	0.796	0.95
	3.78 ± 2.62			2.83 ± 2.60					(−6.27, 8.17
WHZ	−0.16 ± 0.13	0.01 ± 0.13	0.369	−0.16 ± 0.13	0.06 ± 0.13	0.234	0.841	0.840	−0.05
	0.17 ± 0.19			0.22 ± 0.18					(−0.46, 0.56)
HAZ	−0.59 ± 0.06	−0.51 ± 0.06	0.380	−0.62 ± 0.06	−0.65 ± 0.06	0.693	0.367	0.367	0.11
	0.08 ± 0.09			−0.03 ± 0.09					(−0.13, 0.36)

Values are presented as LS means ± SE. Statistical analysis was performed based on the ITT principle. Missing data were handled using MI to generate 20 complete datasets. All results from the imputed datasets were pooled according to Rubin's rules to derive final estimates and *p*-values. (1) Within-group mean changes from baseline were compared using LMMs. (2) Between-group differences in changes were evaluated by analyzing the group-by-visit interaction effect in the model. (3) The LMMs were further adjusted for treatment compliance as a covariate. (4) Between-group effect sizes (estimated mean difference with 95% CIs) were estimated using the MI-based LMMs adjusted for compliance. *$p < 0.05$. **$p < 0.01$. CIs, confidence intervals; CI, confidence interval; HAZ, height-for-age Z-score; IGF-1, insulin-like growth factor-1; IGFBP-3, insulin-like growth factor binding protein-3; ITT, intention-to-treat; LMM, linear mixed model; LS means, least squares means; MI, multiple imputation; SDS, standard deviation score; SE, standard error; WHZ, weight-for-height Z-score.
